# War, forced displacement, and alcohol abuse: experiences and perceptions of war-affected south Sudanese refugee youth living in Bidibidi refugee settlement in northern Uganda

**DOI:** 10.3389/fpubh.2024.1232504

**Published:** 2024-02-05

**Authors:** Godfrey Makoha, Myriam Denov

**Affiliations:** School of Social Work, McGill University, Montreal, QC, Canada

**Keywords:** forced displacement, war, alcohol abuse, South Sudan, Bidibidi refugee settlement, Northern Uganda

## Abstract

Refugees are at high risk of alcohol abuse due to their experiences of structural, physical, sexual, and psychological violence in their countries of origin, during flight, and within host communities. Given the prolonged civil war in their country, South Sudanese have continued to flee profound forms of violence and now constitute the largest population of refugees in Uganda. However, little is known about their displacement experiences, as well as the reality of alcohol use and abuse within refugee settlements. Drawing upon the direct voices of a sample of war-affected South Sudanese young people, this article explores their experiences of forced displacement and their links to alcohol abuse, as well as their perceptions regarding appropriate alcohol treatment interventions for refugees in the camp. A total of 22 semi-structured qualitative interviews were conducted with 14 refugee youth (aged 18–25) alongside eight adult key informants who work with the youth (religious leaders, sports coaches, educators, social workers, and settlement administrators). Using thematic analysis, the study revealed a series of key themes influencing and shaping the high incidence of alcohol abuse among the youth. These included traumatic wartime and migration experiences, family separation, poor prospects, and the ubiquitous availability of alcohol in the settlement. In addition, we show how alcohol operates as a strategic tool for survival for the youth, as well as highlight how these perceptions can help to inform alcohol treatment interventions in the Bidibidi refugee settlement. To our knowledge, this is the first in-depth study of alcohol abuse among war-affected South Sudanese refugee youth in Uganda, addressing a significant gap in the current literature on war-affected youth, forced displacement, and alcohol abuse. We contend that involving youth in the design of interventions can be helpful for culturally sensitive and relevant prevention, treatment, and care in refugee settings. In addition, providing employment opportunities and meaningful engagement for growth through social participation can help to address harmful alcohol use among youth in the camps.

## Introduction

According to the United Nations High Commission for Refugees (1), approximately 89.3 million people were displaced at the end of 2021. The rising numbers of global displaced populations have mainly been attributed to war, armed conflicts, and natural disasters in Afghanistan, Syria, Iran, the Middle East, Ukraine, the Democratic Republic of Congo (DRC), Central African Republic, Gaza, Israel, Somalia, and South Sudan (2). These wars have dramatically altered the lives of children, youth,^1^ and families with profound physical, social, economic, political, and psychological effects. War and war-induced migration have a potent impact on children and youth. In 2017, 420 million children—nearly one in five—lived in conflict-affected areas, an increase of 30 million from the previous year (3). Children are disproportionately exposed to injuries, separation from family, and sexual exploitation, while others are recruited into armed groups. War disrupts healthy child and youth development, causes injury and illness, severs familial, social, and cultural networks, and destroys structures that provide protection and preventive care (4). In 2019, more than half of the forcibly displaced population were children (5). These sudden or protracted crises can have immediate and long-lasting implications, existing in the months, years and decades beyond the original upheaval.

Humanitarian crises such as war and violence feature prominently in the lives of displaced people, where millions of people flee their homes and become refugees. Moreover, researchers (6) have found that over 76% of those forcibly displaced experience protracted displacement, remaining stateless for extended periods. Prolonged crises may lead to negative coping strategies, including alcohol use disorder, a chronic condition. In addition, alcohol brewing and selling can often become a means of obtaining non-food items and a source of livelihood for many families in refugee camps.

Such realities involving war, migration and alcohol use are important to consider among war-affected Sudanese refugees, many of whom have endured decades of war and displacement. Since its independence in 1956, the history of Sudan – a country of 45 million people, of which more than half are children – has been plagued by political and civil strife, fragility, climate change and flooding, and persistent socio-economic crises. One of the deadliest and longest civil wars in African history, war has enveloped Sudanese history and its people. While armed violence has occurred over decades, in December 2013, a power struggle between the president of South Sudan, Salva Kiir, and his deputy, Riek Machar, broke out over an alleged coup (7, 8). Since then, there has been continued fighting and instability along tribal and ethnic lines (Nuer and Dinka), resulting in numerous killings and the displacement of over 2.2 million people (7, 8). Despite the two conflicting parties signing a peace and cease-fire agreement, armed violence in South Sudan has continued. Although many people remained internally displaced, as of January 2016, over 644,000 South Sudanese had fled to neighboring countries, including Ethiopia, Kenya, Sudan, and Uganda. While other neighboring countries have hosted South Sudanese refugees, Uganda has hosted refugees from Sudan since 1955, ultimately leading to the establishment of refugee settlements in Adjumani, Arua, Moyo, and Koboko. Due to the rapid influx of refugees following the 2013 political violence in South Sudan, the government of Uganda established the Bidibidi refugee settlement in 2016 to host South Sudanese refugees.

At the time of this study’s data collection, Bidibidi was and is still the largest refugee settlement in Uganda, with a vast coverage of 250 square miles and a population of approximately 233,959 people. South Sudanese refugees make up more than 60 percent of the total refugees in Uganda (5, 9) and, importantly, over 90 percent of the Bidibidi settlement population, with women and children contributing 85% (10). According to Uganda’s government refugee response monitoring system, 24% of the Bidibidi settlement population comprises youth aged 12–24 (10, 11). The settlement has a mix of people from different South Sudanese ethnic backgrounds, including Kakwa, Kuku Bari, Dinka, Nuer, Madi and Pojulu Bari tribes and are mainly Christians (7, 11). Yumbe district, on the other hand, is predominantly occupied by Muslim locals. The district is also one of Uganda’s poorest districts, with subsistence farming as the primary means of livelihood (12). The Uganda government’s resettlement policies have seen refugees resettled in rural areas (13). With restrictive employment policies and poor climate conditions, most refugees do not benefit from formal employment and agriculture (12). As such, most refugees depend entirely on humanitarian relief and casual labor for everyday survival. However, some refugees who arrived with or acquired some skills through on-site training may be employed by NGOs and other partner organizations.

Studies (2, 14, 15) indicate that populations affected by forced displacement and armed conflict are at higher risk for alcohol and substance abuse. Data show that youth in the settlement have lived most of their lives as refugees (16, 17). Some are born to parents who are refugees and are thus second-generation refugees. (18, 19). Living in a community where there is very little hope and limited upward social mobility can be frustrating for many young people, potentially resulting in negative coping behaviors like alcohol abuse (20). Additionally, researchers (14, 21–24) highlight that war, trauma, and stigmatization can lead to the onset of alcohol use even in communities that once limited alcohol use. Furthermore, in their study of the lived experiences of refugee youth in Somalia, researchers (25, 26) show that refugees in Africa have directly or indirectly experienced loss and anxiety-provoking events, including torture, sexual violence, killings, and abduction, all of which increase the risk for young people to use substances including alcohol. Relatedly, researchers (27) have noted that refugee children and youth in Sabia are at increased vulnerability to harmful alcohol use.

The availability of alcoholic beverages in refugee camps has been observed among the risk factors for the harmful use of alcohol. Authors (17, 28) noted that access to cheap and readily available alcoholic beverages is a significant risk factor for the harmful use of alcohol in marginalized communities. In sub-Saharan Africa, researchers (20, 29) found local alcohol brewing in refugee settlements in Uganda, Kenya, and Ethiopia. In 2006, researchers (27) assessed substance abuse and HIV vulnerability in Kakuma refugee camp in Kenya. The same study showed that locally brewed alcohol called “Changaa” (Maize brew) and “Busae” (sorghum brew) were the most used substances.

There is growing recognition that the harmful use of alcohol has significant consequences for refugees and other displaced populations, including the adverse effects it causes on human organs and tissue, injuries or poisoning, and potential self-harm or violence (30, 31). Alcohol also has an elevated risk of non-communicable diseases (NCDs), leading to an increasing health burden among refugees, including infectious diseases such as HIV/AIDS, tuberculosis, alcoholic liver diseases, heart diseases, strokes, cancers, and gastrointestinal diseases (32). Moreover, the harmful consumption of alcohol in refugee settlements has been associated with, but not limited to, increased domestic and family violence, risky sexual behavior, suicide, assaults, school dropout, and road accidents, alongside several health risks among young adults (20, 33). The consequences of alcohol abuse are far-reaching and range from individual health risks to implications for family, friends, and the larger society. Despite these consequences, alcoholic beverages have remained widely available in refugee settings (16, 17, 34).

Although approximately 86 percent of the world’s refugee population resides in low-income and developing countries (16), few studies have explored alcohol use among refugees in the Ugandan context. Most existing literature has focused on high-income countries (17, 35, 36), and little is known about South Sudanese refugees living in northern Uganda. An important exception is a systematic review of harmful alcohol use among civilian populations affected by armed conflict in low- and middle-income countries (37). This research found a significant association between trauma exposure and harmful alcohol use among Internally Displaced Persons (IDPs) in northern Uganda. The specific type of traumatic events identified included abduction, torture, rape, and imprisonment (38).

The use of alcohol is widely recognized as having severe physical, social and health consequences. In refugee camps, harmful alcohol use can further diminish an individual’s quality of life and increase morbidity and mortality rates. Existing literature highlights significant socioeconomic and health needs for refugee youth, making it crucial to investigate the intersections of forced displacement and alcohol abuse in the Bidibidi settlement, which is the goal of this paper. To our knowledge, this is the first in-depth study exploring alcohol use among war-affected South Sudan refugee youth in Uganda, addressing a significant gap in the current literature on war, forced displacement and alcohol abuse. Moreover, privileging the perspectives of young refugee youth can develop effective interventions to address alcohol use and misuse in vulnerable refugee communities. To that end, we draw on the direct voices of a sample of South Sudanese youth and key informants to explore the experiences of alcohol abuse in the context of war-related displacement.

## Methods and materials

This article is part of a more extensive qualitative study (39) that sought to gain first-hand information on forced displacement experiences and alcohol abuse among war-displaced youth. Hinged on the critical realism paradigm (40), a case study design was adopted to enable the researchers to gain broader, in-depth information along with the symbolic practices, meaningful beliefs and emotions inscribed in daily interactions. The population for this study was drawn from South Sudanese refugee youths living in the Bidibidi settlement in northern Uganda, as well as key informants who worked with them.

Ethical approvals for this study were received through the McGill University Research Ethics Board III. This study was further approved by the Office of the Prime Minister (OPM) through the Refugee Commissioner General in Uganda, and the Bidibidi refugee settlement Commandant Officer provided approval before data collection in the settlement. All the required steps were taken to ensure participants were thoroughly informed about the study objectives and the protection of the information they provided. Additionally, participation in this study was voluntary and informed consent of the study participants was sought before and during interview sessions. The first author is a trained social worker and was able to address distress directly when it arose during the interview.

To be eligible for inclusion in the study, youth (both young men and women) of South Sudanese origin were required to have lived in the Bidibidi refugee settlement for at least a year before the scheduled date of the interview. Regarding age, the youth aged between 18 and 25 years were accepted based on the majority age in Uganda. Key informants actively working with the youth in the settlement for 6 months or more by the time of data collection were eligible for selection to participate in the study.

To recruit participants, the first author worked closely with social workers from the Office of the Prime Minister (OPM) in Bidibidi Base Camp. Recruiting participants during the COVID-19 pandemic, when the data collection occurred, was a significant challenge. To overcome this, the assistance of a social worker who had existing solid relationships with the youth living in the settlement and key informants was sought. The social worker actively engaged youth who varied in age, gender, wartime experiences, and time in the camp. Given their knowledge of the youth in the camp, the social worker also helped to assess whether the youth were well enough to participate and avoided engaging youth who might be at risk of experiencing undue stress or mental health problems as a result of discussing issues related to war, migration and alcohol consumption.

In addition, the social worker shared recruitment materials with both youths and key informants and ensured that they understood the study’s goals. The social worker then collected the contact details of interested participants and provided them to the researcher, who followed up. Given the violent and distressing events that these young people experienced during the war, in flight, and upon resettlement, and the sensitivity of research, the first author independently conducted an initial phone or WhatsApp screening for current distress to exclude potential participants with emotional difficulty. Only the youth who passed the phone screening and could comprehend the English language were eligible to participate in the study. To assess participants’ mental health, the Refugee Health Screener RHS-15 was employed. Participants were asked the following three yes/no questions during the phone screening:

1. Are you currently receiving any professional mental health services?
2. Do you have difficulty remembering past events (Cognitive challenges)
3. Do you have sleep disturbances, feeling sad, angry, anxious, or depressed when discussing your past experiences?

Those who answered “yes” to any of the three questions were excluded from the study.

A total of 22 participants, including 14 refugee youth aged 18–25 and 8 adult key informants aged 26–65, were purposively selected for interview. A semi-structured interview guide was developed and used to collect data from the youth. Although the interview guide exposed all the youths to similar questions, non-active alcohol users were probed to provide more information on the protective mechanisms they used to avoid problems with alcohol. In contrast, active alcohol users offered more insights into the risk factors and the influence of alcohol use and abuse. A semi-structured key informant guide was developed and used to collect data from the key informants, who were community leaders (Local Council officials and law enforcement officers) and Bidibidi settlement staff members (refugee welfare council officials, social workers, sports coaches, educators, health workers, and counselors) working with the youth at the community level.

Drawing on their personal, professional, and community experiences working with the youth on diverse programs, key informants were able to provide deeper insights into the war, displacement, and alcohol use. All interviews were audio-recorded and transcribed verbatim by the first author. All identifying information shared by the participants in the audio recordings was removed during transcription. To support study participants, the social work department in the camp was available to provide debriefing support for participants. In addition to providing emotional support, the department offered various other services, including educational support, health services, and recreational services. The study participants were provided information about waiting lists and the exact nature of services. Risks to key informants and settlement staff members were minimal as they were interviewed in their professional capacities.

The interview transcripts were coded using Nvivo 12 software. Transcripts were read multiple times to draw codes related to the experiences of forcefully displaced South Sudanese youths with alcohol abuse in the Bidibidi settlement. This was followed by inductive approaches, including identifying conceptual phrases used by the participants to generate categories. A total of 32 categories were then grouped to capture the complexity and diversity of the data. Categories were then merged into 22 themes and 12 subthemes, all supported by direct quotes from individual study participants. Examples of themes and subthemes that emerged from the data included alcohol as a coping mechanism, alcohol use and cultural breakdown, and the use of alcohol as a means of livelihood and socializing. To ensure member-checking, the themes were reviewed by the local social worker and the key informants, alongside youth who did not participate in the study but had similar experiences, to verify that the emerging themes resonated with them.

There are two main limitations we note in this study. First, the study was conducted during the COVID-19 pandemic, and the restrictions could have affected other potential participants for in-person interviews. Secondly, as with all self-report data, the realities and limitations of memory and self-disclosure were not considered, although we acknowledge some of the violent and distressing events that these young people experienced during war, flight, and upon resettlement. Therefore, it is conceivable that, occasionally, some of the participants may have concealed some anxiety-provoking aspects of their stories. Lastly, this is a qualitative study with a small number of participants. Given the sample size, the findings cannot be generalized to all refugee youth living in the Bidibidi refugee settlement.

## Findings

### Table showing demographic characteristics of participants

The table above represents the demographic characteristics of 22 participants comprising 14 refugee youth aged 18–25 years and eight adult key informants aged 26–65. The youth arrived in Uganda between 2016 and 2017 following an escalated tribal conflict in South Sudan. Key informants comprised local community leaders, social workers, refugee welfare officials, community mobilizers, and religious leaders who work closely to administer and provide services for refugee youth in the Bidibidi refugee settlement (Table 1).

**Table 1:** Demographic characteristics of the study participants.

Participants	Age in years	Religion	Education status	Role with refugees	Country of origin
Refugee Youth Male 1	25	Anglican	Primary Education	Volunteer	South Sudan
Refugee Youth Male 2	24	Anglican	University education	Community Volunteer	South Sudan
Refugee Youth Male 3	25	Catholic	Secondary Education	Not in School	South Sudan
Refugee Youth Male 4	25	Catholic	Primary Education	Not in School	South Sudan
Refugee Youth Male 5	25	Catholic	Secondary Education	Not in School	South Sudan
Refugee Youth Male 6	23	Catholic	Secondary Education	Schooling	South Sudan
Refugee Youth Male 7	22	Muslim	Secondary Education	Schooling	South Sudan
Refugee Youth Male 8	24	Anglican	Secondary Education	Not in School	South Sudan
Refugee Youth Female 1	24	Catholic	Secondary Education	Community Volunteer	South Sudan
Refugee Youth Female 2	19	Anglican	Secondary Education	Schooling	South Sudan
Refugee Youth Female 3	21	Catholic	Secondary Education	Schooling	South Sudan
Refugee Youth Female 4	19	Catholic	Primary Education	Schooling	South Sudan
Refugee Youth Female 5	22	Muslim	Primary Education	Not in School	South Sudan
Refugee Youth Female 6	23	Anglican	Primary Education	Schooling	South Sudan
Key Informant Male 1	34	Anglican	Diploma	Religious Leader	South Sudan
Key Informant Male 2	43	Catholic	Diploma	Refugee Welfare Official	South Sudan
Key Informant Female 1	26	Born Again	Primary Education	Community Mobilizer	South Sudan
Key Informant Male 3	45	Born Again	Secondary Education	Community Leader	South Sudan
Key Informant Male 4	28	Catholic	University Education	Social Worker	Uganda
Key Informant Male 5	65	Muslim	Primary Education	Community Leader	Uganda
Key Informant Male 6	30	Catholic	University Education	Social Worker	Uganda
Key Informant Male 7	41	Muslim	Diploma	Refugee Welfare Official	South Sudan

Drawing upon the direct voices of war-affected South Sudanese young people and adult key informants, this article presents three themes and eight subthemes that emerged from the youth and key informant interview data. These include alcohol as an anesthetic, alcohol as a booster and tool for survival, and the youth and service providers’ perspectives on treatment and intervention. The subthemes that emerged include alcohol and its links to war and displacement experiences, alcohol as a means of coping with loss and separation from family, alcohol as a means of dealing with cultural breakdown and fragmentation, alcohol as a means of coping with perceived poor prospects among the youth, alcohol as a source of economic survival, alcohol as a tool for social interaction and relationship building, alcohol as a means to cope with stress and loneliness, and alcohol as a perceived cure for the COVID-19 virus. Participants underscored the widespread production of local brewing in the settlement and observed that people were increasing their alcohol consumption in the camp. Participants reported that in 2016, when they came to the camp, there was zero alcohol production in the settlement. However, they noted that people now tended to drink more, the longer living in the camp, and as their individual and collective hardships continued. Participants perceived that as the crisis in South Sudan has become protracted – “a marathon rather than a sprint” – alcohol brewing and consumption has also become chronic and used not only as a coping mechanism but also as a source of livelihood for displaced families engaged in the brewing business. The following section addresses these multiple themes in greater detail.

### Alcohol as an anesthesia

Participants often narrated that youth drank alcohol as a negative coping strategy to deal with the crisis and its related grief, sorrow, hardship, and loss. Participants reported that some youth consumed alcohol to numb and forget the psychological stress and trauma, fear, experiences of war and their prolonged stay in the refugee settlement.

#### Alcohol and its link to war and displacement experiences

Both groups of participants reported that many youth in the settlement were using alcohol to forget about past wartime traumatic memories in South Sudan. The violence that participants and their family members had endured in South Sudan, as well as during the migration journey, was often linked to alcohol consumption. Alcohol was reportedly used as a coping mechanism in the aftermath of such profound crises and hardships. As this youth participant explained:

*… killing in South Sudan was too much, and we decided to leave with my brother. We suffered from hunger on the road, and even water was a problem. On our way, my brother was shot dead. So, when I got here, I found life was difficult and it was my brother who used to help me. By the time we came here, there was nothing I could get, and I started thinking of my brother. I said, if my brother could be there, then I should not have this difficult life. So, because my brother was killed, it gave me too much stress, and I started drinking alcohol*. [Refugee Youth Male 8].

These key informants shared their perspectives on the link between forced migration, violence, and alcohol consumption among the youth:

… *most of these children you see around are fostered children. Many are UAM they are unaccompanied. UAM means unaccompanied minors. Those are children who ran when they lost their relatives when they lost their parents, making them flee because of fear of being also killed as it happened to others. So those are the conditions that made people come. You see some of the family members are being killed, then you see all your properties are being destroyed or collected, and you remain there alone. Automatically, to stop thinking about that, they will be forced to drink alcohol*. [Key Informant Male 2]

*…most of the young people from the refugee side have involved themselves in too much drinking due to the following reasons. One, the majority of our children have not gone to school. There is a high rate of illiteracy, OK? This has left many of our youths unable to make creative minds and be innovative in thinking about what they can do to make themselves sustainable or reliable. Then two, trauma, you know, when people were running, people lost a lot of their properties, and of course, when you are a visitor in another land, you will never be the same. For quite a period, you will be thinking about what you have been having, and you will be thinking about how you will recover now. You will get confused and feel a lot when you see the limited opportunity*. [Key Informant Male 3].

#### Alcohol as a means of coping with loss and separation from parents/guardians

Participants reported that the stress from separation was a contributing factor to alcohol abuse after resettlement in Bidibidi. We observed that many young people lived in the settlement without their parents. When asked about the whereabouts of their parents, some youth reported that they came to the Bidibidi settlement without any parent or guardian. In other cases, the youth said their parents had returned to South Sudan and left them in the settlement. Participants linked their loss of family and lack of parents and guardians to alcohol consumption:

*But there are also some youth, let’s say, who are orphans; they don’t have someone like a relative in the camp; others left their parents in South Sudan, and others are total orphans; they do not have any parents. So, because of hardships, instead of focusing on what will change their lives, they will just get engaged in drinking alcohol because they think it is the last option for them to do*. [Refugee Youth Female 2].

*Yes, I even have one of my friends. We reached out to him as a team and tried to talk to him. The question was, he told us we should not waste our own time because of him. He said he has lost many things in his life; he wants to take alcohol so that he can also lose his life like he has been losing his people. Then we tried to tell him that maybe he would be the root of his family. So, we try to advise these youth. [Some] understand while others do not understand. We have one of our friends, also, who has lost both parents on the road to Uganda. And since we came here, all the time he was taking alcohol*. [Refugee Youth Male 6].

Participants asserted that alcohol abuse was not only associated with the lack of parents/guardians but also the loss of parental guidance and socio-economic protection within the settlement itself. The parents’ failure to provide for the family, particularly for the youth, reportedly resulted in an expectation-reality gap leading to socio-economic loss and family breakdown. As this key informant explained:

*… [Fathers] are supposed to head the family, but here is a situation where these men cannot provide anymore. Their hands are tied up; almost everything has failed for now. I should say the economic aspect traumatizes them, so alcohol is used so that the wives, children, or relatives cannot ask them. They will know this is now a drunkard, So one factor is they are doing it to dodge responsibilities at home, which is true*. [Key Informant Male 6].

#### Alcohol as a means of coping with cultural fragmentation & breakdown

Many participants described in depth how enduring war in South Sudan, flight across the border into Uganda, and their protracted stay in the Bidibidi settlement had disrupted and fractured their sense of culture, identity, and traditional ways of living. The South Sudanese refugees had lost not only family members, relatives, and neighbors but also those who fled the South Sudanese war at a young age and were growing up in the settlement reported losing their traditional South Sudanese culture. These youth explained the link between cultural loss and fragmentation with alcohol use and abuse:

*… as people were fleeing the conflicts from South Sudan to other places, people were mixing and copying from each other’s culture. Here in the settlement, we have many tribes, many of whom are alcohol prone even when they were in South Sudan before the war. They also brought that habit here in the settlement*. [Refugee Youth Male 7]

*… in the Kuku culture, the child becomes an adult at 20,21, or 22 years or more. If the child at this age is caught drinking alcohol, they will be seriously punished by their parents or even the community. But today, as we are speaking here in the settlement, we see our children taking alcohol even at the age of 18 years because they were told that at that age, they are mature enough to do whatever they want. Living in the settlement has destroyed our children. If we had brought up our children according to our culture, we could have well-behaved children*. [Key Informant Male 3]

Coming to the Bidibidi refugee settlement in Uganda meant adapting to a new context and learning a new language and culture, all the while facing long-term unemployment. Many participants articulated that although they had rebuilt some sense of community, most youth reported feeling broken, fractured, and lost. This youth describes how the breakdown of culture is both a result of war-induced migration as well as a key component in the inability to prevent or address alcohol abuse:

*When we were in South Sudan, it was the elders who were drinking alcohol and beer. Traditionally, if a child is caught drinking alcohol, they will be seriously beaten by the parents. And if, as a young youth, you taste the alcohol, you will be afraid because you are aware that you could be seriously punished. I know not all are drinking alcohol, but settlement has destroyed our culture, and no one cares. If we had grown up our children according to our culture, we could have well-behaved children and not drink too much like this*. [Key Informant Male 4].

Both key informants and youth reported that the problems related to alcohol use and abuse, such as family neglect, family violence, suicide, assaults, and sexual abuse in the Bidibidi settlement, affected the whole community of South Sudanese refugees in the camp.

*… once the youth get involved in drinking alcohol, they lose the ability of responsibility and honesty and become careless with their lives. They get involved in unprotected sexual affairs under the influence of alcohol and intoxication. The other significant consequence is suicidal acts. We have cases of attempted suicide, and we have successful suicide, which has happened here in this settlement due to alcoholism. Some people have died after repeated abuse of alcohol in the settlement here* (Key Informant Male 1).

This young woman described the gendered realities, implications, and insecurity of life in the camp and the links to alcohol:

*… And once you are caught alone, maybe you are two, and for those boys, they are many in a group, they may turn to rape you and, at times, beat you up. That is the problem we have seen with alcohol; it is affecting everyone, even young children below 18 years of age* (Refugee Youth Female 3).

#### Alcohol as a means of coping with perceived poor future prospects

Youths in the Bidibidi settlement reported using alcohol as a result of boredom, isolation, and hopelessness in the settlement. For the older youths, there were few educational and employment opportunities in the settlement, and the youth reported spending much of their time simply hanging out in the trading centers with nothing to do other than playing cards and drinking alcohol. Participants said that the war and resulting displacement fostered feelings of powerlessness and hopelessness, which reportedly had a critical impact on alcohol use. This key informant explained:

*… when people were running, people lost a lot of their property, and of course, when you are a visitor in another land, you will never be the same. For quite a period, you will be thinking about what you have been having, and you will be thinking about how you will recover now when you see the limited opportunity; that is when you will get confused, and you will think a lot. For you to reduce your thinking, you will have to drink [alcohol]* [Key Informant Male 2].

#### Alcohol as a means to cope with stress and loneliness

Participants maintained that past wartime experiences and associated memories did not solely cause alcohol use but also the settlement situation itself, which reportedly caused distress, boredom, and loneliness among the youth:

*… actually, spending a week here is not something easy because we, the youth, are idle, and there is nothing that we can do. You find that if the day starts like this, we shall just be loitering around, playing cards, drinking alcohol, and playing dominos (gaming machines), and there is no other help we can get from that. And life is not all that fine* [Refugee Youth Male 8].

Participants often reported increased stress related to their education, employment, acculturation, weather conditions, housing, food, language, and health in the camp. Participants reported that disruptions in resettlement and trouble connecting with their host community invoked feelings of loneliness, making many young people feel socially isolated and marginalized:

*Well, at first, it was hard because people in this community were insulting us by saying, you South Sudanese can not stay on our land and destroy our trees because there were very many South Sudanese coming in Uganda. I felt that I was not welcome, I felt like I had nowhere to go, my life was ruined, and I did not know why I was still living*. [Key Informant Male 1].

Alcohol use and abuse were perceived as a way of escaping, dealing, or coping with the multiple and complex stress-related events that occurred in the settlement.

*I want to say that the rate of youth taking alcohol is high, particularly in my zone. One could be due to a lack of parental guidance. You know, most of the people who are here in the settlement are women and children. Most men were left behind for one reason or the other. Then you know these women are regarded as weak, and these youths tend not to respect them, leaving a gap for these people. Then, two, it could be unemployment. Some youths have gone to school, and here they are redundant. Some have documents, and getting a job here is very difficult, which also contributes to the use of alcohol. The other could be peer group influence; you know, in this world, we have good and bad friends, so that one could contribute*. [Key Informant Male 3]

### Alcohol as a booster and tool for survival

Participants reported that due to the prolonged crisis and extended stay in the settlement, some people turn to alcohol brewing as a business. Participants also reported that young people drink alcohol to get energy, boost their confidence, feel more positive or enjoy the company of friends. Both sets of participants reported limited formal employment opportunities in the camps, and the soils do not support crop farming. According to participants, they were brewing alcohol thus provided a means of livelihood and economic survival, a means of socializing and building relationships, a way to contend with stress and loneliness, as well as a “medicine” for preventing or curing COVID-19. This section addresses these themes in greater detail.

#### Alcohol as a tool for economic survival

Participants described that some youth in the settlement were engaged in local alcohol [Nguli] brewing as a source of livelihood. These youth describe locally-made alcohol, its accessibility, and its use as a form of economic survival:

*As we receive food, we have been selling it to buy charcoal, pay some fees for children in school such as development fees, and buy books and soap, but as you know, the food that is left is little, and hunger, therefore, affects us ….so we starve until the next food is distributed … But now we brew this alcohol, and we sell this alcohol to buy what is not provided to us like soap, milk for the children*. [Refugee Youth Female 3]

*Locally, here we have what they call “Nguli,” made from cassava and yeast, made from millet or sorghum. It is a bit risky but locally made here. It is the most common and the cheapest. But we also have others which are factory made, there is one called Coke Gin, Vodka, Cheza, Ola, the Best wine, Jonnies is also cheap, and these people [youth] want to consume alcohol that makes them drunk as soon as possible or as quickly as possible*. [Refugee Youth Female1].

#### Alcohol as a tool for social interaction & relationship building

Alcohol use and abuse among the youth were perceived as a gateway and facilitator of social interactions and friendships with peers. Many young people articulated that sharing alcohol was not only a way of having fun with friends but also helped to create relaxed environments to find support in their shared experiences. Drinking alcohol was associated with social experiences and a means to help build and strengthen new friendships. This youth explained the link between moderate alcohol consumption and family and community building in South Sudan:

*In South Sudan, alcohol used to be consumed by elders around evening hours. Alcohol was consumed in moderation and for socialization among family members, relatives, and friends*. [Refugee Youth Female 1]

Shared beliefs, norms, and expectations also played a significant influence on alcohol use and abuse among young people in the settlement, with some participants reporting that declining to share alcohol when offered by a friend was considered anti-social and inappropriate:

*You know how people are working. I am a construction worker, and you spend the whole day standing, and by the time you return home, you are already tired and feeling pain. If you call your friends, they will tell you they want something to energize them. Of course, when they call you to come to the trading center, they will buy some alcohol, and you can not refuse to take it because they feel bad. It is not good to refuse when a friend buys you a drink*. [Refugee Youth Male 5]

#### Alcohol as a perceived “cure” for the COVID-19 virus

Many participants perceived alcohol as serving a medicinal purpose and that alcohol had a functional use. The participants mentioned that alcohol was good for their health – even acting as a cure for the COVID-19 virus. Some young people reported that they drank alcohol to address the boredom of the lockdown and to “sanitize” their bodies from COVID-19:

*Now, they are increasing, and because of COVID-19, the cases are growing too much; the students even start drinking alcohol because of this lockdown. So, because of this lockdown, there is no school, and others are saying let us drink this alcohol to sanitize our bodies. They say that alcohol kills the COVID-19 virus*. [Refugee Youth Female 6]

*Then, due to this issue of COVID-19, it is difficult since we have been locked up and there is no school; we are finding life difficult. That is why most of the youth ended up giving up and saying if this is their life, they better do what they can do. That is why most of them end up drinking alcohol, and some of them are even smoking opium and cigarettes*. [Refugee Youth Male 9]

### Youth and service providers’ perspectives on treatment and intervention

This study explored youth perspectives on the availability and accessibility to alcohol use services and intervention programs directed to the youths in the Bidibidi refugee settlement. Both youth and key informants often reported that there is no specially trained personnel in any of the partner organizations to coordinate or implement services related to alcohol use among the youth in the Bidibidi settlement. Similarly, both sets of participants also reported that there are no specially designed programmes and interventions to manage the health and social consequences of harmful alcohol use among abusers and those indirectly affected in the Bidibidi settlement:

*Specifically, services targeting youth who are drinking alcohol are not there; we don’t have it, but it is general protection. We have IRC (International Rescue Committee), World Vision, OPM, and UNHCR, where alcohol is incorporated, but as a specific organization, looking into youth who are engaged in drug abuse is not there. In the settlement here, we used to have CARITAS. CARITAS Uganda used to come to the community for meetings with parents and children, and even at schools, they could visit and advise children on the dangers of alcohol. Still, since they left many years ago, no partners have talked to the youth or counselling them that they can do this and that*. [Key Informant Male 2]

The exception mentioned was for emergencies, especially where the individual is experiencing a life-threatening condition due to alcohol. Both youth and key informants highlighted that when an individual had a health crisis or complication due to alcohol abuse, it was commonly family members, relatives, and, at times, significant others who attempted to seek help from the Bidibidi Health Centre. Participants highlighted that there were no designed rehabilitation programmes or services for alcohol-dependent youth:

*Let me say no organization is working to stop alcohol. But here, let me say that RWCs (Refugee Welfare Committees) are the ones who help the children during emergencies. If they see the way youth are behaving, others who would fight, they assault themselves, or when the person collapses, they will take them to the health center. But it is the family members and friends, let me say, peers, help the youth many times*. [Key Informant Female 1]

Both sets of participants reported that alcohol use campaigns were not conducted regularly, consistently or in a coordinated manner. They also mentioned that there are few materials available to the youth on alcohol use-specific issues. Refugee youth also reported that they struggle with stigma from self, family, friends, the host community, and health care systems. The youth perceived that given that the majority of host communities belonged to the Muslim faith, they condemned alcohol use. Participants reported that the stigmatization from the host community further destabilized their lives, as they felt shame to seek help at health facilities outside the settlement. These participants explained:

*You see, I cannot go to the hospital for treatment because people will laugh at me; alcohol is not sickness, and they will say, look at this drunkard because most people in Bidibidi [host community] are Muslims. I feel ashamed to go to the clinic in the host community*. [Refugee Youth Male 1]

*One is that at times the youths don’t attend our meetings. They are like it is not their concern now, and we blame them. They fear attending community health meetings and even going to the health center. Then two, especially those schoolgoers, don’t have time to attend and meet us, but we also have a strategy in our simple leadership structure. We have a leader for the youths from various levels, and at times, when we organize a meeting, we spearhead these youth leaders and empower them to talk to them*. [Key Informant Male 4]

## Discussion

### Alcohol use/misuse among war-affected youth

Our findings suggest that alcohol use and abuse among South Sudanese refugee youth are shaped by a multitude of complex factors related to the war in South Sudan. Interviews uncovered that alcohol consumption is an essential strategy for the youth to attempt to forget about the war and its related grief, sorrow, stress, loss, and the multiple hardships attributed to prolonged stay in the camp. Alcohol consumption was also used as a form of anesthetic to cope with traumatic wartime experiences and the challenges of flight and resettlement. These experiences include mass and indiscriminate killings, torture, abduction, burning of houses, looting and destruction of property and violence. Findings align with existing studies that have noted the use of alcohol as a coping strategy to deal with stress (21, 37, 41, 42).

The findings also indicate that war trauma and forced displacement exacerbated vulnerability to harmful alcohol use among the young people in the settlement. These findings are consistent with the existing literature (36, 37) that has found that continuous exposure to traumatic events is associated with alcohol use. Additionally, researchers (35, 43–45) have illuminated that refugees who experience war or conflict-related trauma are at greater risk for poor mental health and alcohol abuse. This study has revealed that some youth consume alcohol to cope with and forget war-related atrocities. Findings also show that the youth drink alcohol to cope with the daily frustration from settlement challenges such as illness and inadequate basic needs like food, shelter, and education, but also to “fit in” with their peers who drink alcohol. This is in line with other research on Somali refugees (17) that found that young people use substances such as Khat, Tobacco and alcohol as an essential way to build social bonds and as a representation of their anger, frustration, and resistance to failed protection from the Kenyan and Somali communities.

The findings also demonstrate that alcohol is used as a booster and tool for survival in crises. Alcohol brewing serves as a source of livelihood for many families in the settlement. Some women brew and sell alcohol to buy non-food items such as clothes and pay hospital bills and school fees for their children. Alcohol is also associated with socialization, pleasure, and fun among the youth. These experiences are centered around bars and drinking outlets in the trading centers within the settlement. This is similar to a study (14) among Congolese Babembe male refugees resettled in camps.

According to both sets of participants, protracted stay in confined refugee settlement conditions and the realities of boredom, loneliness, and poor prospects contributed to alcohol abuse. In addition, participants reported extremely stressful and prolonged settlement issues such as lack of employment, lack of education, and struggles associated with acculturation, extreme weather conditions, inadequate housing, food insecurity, and language barriers. These findings support previous evidence that experiences of severe resettlement challenges may predispose young people with refugee backgrounds to a wide range of poor health and social behavioral outcomes (19, 43). Additionally, researchers (9) have reported the association between a prolonged stay in harsh refugee camp settings and substance abuse among Congolese refugees.

Findings also reveal the local brewing of alcohol, alongside the availability of numerous drinking establishments in the Bidibidi settlement. Similarly, researchers (16, 17, 37) have noted that refugee communities are becoming increasingly affected by home-brewed alcohol consumption. Additionally, research (19, 43) has illuminated the prevalence of a culture of alcohol consumption in refugee camps. Moreover, as found in this study, alcohol use and its associated disorders can be highly stigmatizing, whereby moral deficiency or lack of self-control and willpower are attributed to affected individuals. This challenges youth seeking help, ultimately affecting treatment-seeking and recovery efforts. In a similar vein, in a study of Karen refugees, researchers (22) established that rebuilding community structures and bonds destroyed by conflict and displacement were essential ingredients to address harmful alcohol use in a refugee setting. Thus, building a supportive network of peers is necessary to support access to services in refugee settlement contexts and reduce shame and stigma.

Participants indicated that alcohol use and abuse were linked to loss and cultural breakdown. Research has underscored that refugee children are at substantially higher risk than the general population for a variety of specific psychiatric disorders related to their exposure to war, violence, torture, and forced migration (46). Moreover, children who are separated from their families pre- or postmigration are said to be at increased risk of psychological and social challenges (47). In this sense, the mental health of refugees is powerfully influenced by war-related violence and loss combined with the conditions they encounter in route to and within their host contexts. It is thus not particularly surprising that youth in the Bidibidi settlement are struggling with issues of loss, separation, and the fallout of war-related violence. It does, however, highlight the need for more excellent policy and service attention to provide those living in refugee camps with greater access to vital mental health support to address the violence and upheaval that they have endured.

### Implications for intervention services, policy and future research

In the absence of prior published studies on the experiences and perceptions of war and forced displacement realities of alcohol abuse among South Sudan refugees in Uganda, this study provides a baseline for further research and contributes to policy and practice knowledge. As in many African countries, alcohol use is part of many cultural, religious, and social practices in Uganda (48). Alcohol consumption is a common characteristic of Ugandan social life among the general population (48, 49). For South Sudanese refugee youth in northern Uganda, it is thus reasonable to argue that drinking alcohol is part of the acculturation process (14, 19). However, harmful alcohol use can lead to short-term and long-term negative consequences for war-affected refugee youth, making it essential to prioritize policy attention. To reduce alcohol use and abuse in refugee settings, cost-effective policy options such as regulating alcohol availability, restricting public drinking and regulating alcohol advertisements in refugee camps can be implemented (50). Age restrictions and greater taxation of alcohol are also effective measures to curtail harmful alcohol use (51).

Most approaches to refugee psychosocial support, including alcohol use services and treatment options, have been dominated by perspectives from the Global North, which center on individualistic approaches to practice, intervention, and treatment (52). Additionally, responses and services may rely on a biomedical model, overemphasizing trauma and adversity and overlooking critical cultural meanings and idioms of trauma, distress, and recovery (53). However, cultural literacy is essential in crises and context matters (54). Eurocentric approaches to care are often adopted without question, which can further marginalize refugee youth, potentially leading to their disconnection from formalized treatment and intervention services (55, 56). Researchers have suggested (57) that: “Contextual factors, including cultural, political and socioeconomic environments, influence explanatory frameworks for mental health problems in children, which in turn influence how distress and impairment are experienced and how symptoms are interpreted in a given society.” Any intervention or prevention strategy developed for youth in the Bidibidi settlement must be grounded in their unique cultural and social contexts and include efforts to interpret the meaning of young people’s expressions of distress and coping. Understanding young people’s explanatory models of “(un)wellness” can help to tailor intervention practices better to galvanize their individual and collective resilience, strengths, and capacities. In addition, drawing upon and fostering South Sudanese cultural activities, celebrations, and networks within the camps, whether formal or informal, could help develop intervention practices and maintain cultural identity and rootedness.

There is a lack of documentation on the importance of service providers, such as social workers, and their role in ensuring refugees in Uganda have access to essential services. However, their work is vital. Many service providers are a part of the Refugee Eligibility Committee (REC). In contrast, others work with Non-Governmental Organizations (NGOs) to help with measures such as first aid, food distribution and health promotion initiatives. Local service providers must be prepared and committed to addressing the vulnerabilities of refugees and their families and national and international policies for social services provision. Moreover, it is essential to strengthen the resilience capacities of refugees to develop and nurture existing informal care systems that assist in meeting their health needs. Our study found that poor economic prospects and survival were the primary reasons why youth used and sold alcohol in the settlement. Therefore, policymakers should focus on long-term economic empowerment and youth employment to address this issue. Additionally, education on the short-term and long-term implications of alcohol use and abuse, including awareness raising and community sensitization regarding alcohol consumption, is crucial. It is also important to correct misinformation that alcohol can cure illnesses such as COVID-19. Our findings on why youth use alcohol as a coping mechanism can help inform policymakers on decision-making, resource mobilization and allocation for alcohol use services and treatment among refugee populations.

Moreover, in addition to good interpersonal skills to work with individuals and groups of refugees, there is an underlying expectation that service providers should exercise leadership in the planning and delivery of services. For example, identifying the health needs of refugees, mainly related to alcohol use and misuse, communicating with potential service providers, providing accurate information to refugees, acting as liaison officers in the community, and working as health care advocates can facilitate promptly delivering required health needs. Direct engagement with refugees allows service providers to understand refugees’ lived experiences better and are better positioned to research issues, including health, culture, and other social determinants of health and wellbeing. In line with this, service providers must reflect on a culturally attuned framework to the health needs of refugees. Attaining these policy and practice goals continues to be a challenge as health services in Uganda face several difficulties, including underfunding and understaffing. The support for humanitarian assistance and protection programs for refugees also face similar hurdles. Underfunded health programs inhibit not only the design and delivery of health services to refugees but also exacerbate their vulnerability to the risk of consequences. Accordingly, shame and stigmatization from self, family, and friends, as well as the host community, were reported as barriers to care, preventing affected youth from attending alcohol treatment services. Notably, youth can receive positive messaging and personalized interventions from service providers. Still, if they return to or live in a context where stigma prevails, all this work can be stunted.

Developing effective intervention and prevention strategies requires us to be involved and learn from the knowledge and experiences of young people who have been affected by violence, loss, and economic marginalization, especially refugee youth in camps. Their unique perspectives and experiences can help us better understand how alcohol use can become a pathway to addiction for them. Future research, policies and practices must be informed by young people’s insights and proposed strategies to address alcohol use and its long-term implications. However, we must also understand that the lives of young people are not independent of their surrounding families and communities that encircle them (4). Thus, any future intervention policy and practice should also consider the perspectives of their families and communities. Moving away from an individualistic approach, we must engage families and communities as active protective mechanisms that can reduce the devastating impacts of war, conflict, displacement, and resettlement through support and protection for the youth.

## Conclusion

To our knowledge, this is the first in-depth study to explore alcohol abuse among war-affected South Sudanese refugee youth in Uganda. This article reveals a wide range of historical, political, cultural, familial, individual, community, and geographical factors that are perceived to influence alcohol use and abuse among war-affected South Sudanese youth living in the Bidibidi refugee settlement in northern Uganda. These factors are essential in stimulating further discussions to counter, buffer, and address the harmful use of alcohol among refugees. The findings show that alcohol abuse among South Sudanese refugees is precipitated by war and conflict experiences before, during flight, and after resettlement. Experiences of trauma, violence, separation from family, breakdown of cultural identity, poor prospects, economic marginality, and easily accessible and inexpensive alcoholic beverages within the Bidibidi refugee settlement are perceived main risk factors for alcohol use and abuse among the young population.

The data further reveal that many youths appear to be caught in a cycle of increasing alcohol consumption, which is influenced by the trauma of war, forced displacement, and the conditions of their refuge. Moreover, the youth highlighted the stigma and shame associated with their alcohol addiction, alongside the fear of seeking support at community health clinics. To tackle youth substance abuse in refugee camps, it is therefore vital to not only address past traumas that appear to drive young people to alcohol use but also the fear and shame that prevents them from seeking help. Addressing alcohol use and abuse among forcefully displaced populations requires a concerted effort from multiple sectors while involving several layers of engagement. This article contributes to an emerging understanding of how South Sudanese refugees perceive alcohol use and abuse in their war displacement experiences, which can provide helpful insights for tailoring interventions to the needs of refugees with alcohol use and abuse problems. Applying group work skills and community work approaches, service providers can liaise between refugees, the government, policymakers, and local authorities to identify priority needs and the necessary resources to meet refugee health needs. These findings may contribute to improved resources and support for alcohol abuse treatment services in post-conflict and humanitarian settings in Uganda and beyond, particularly for young people in similar situations.

In line with the Global Strategy for the Health of Children and Adolescents 2020–2030, improving education, water, harmful alcohol use, and hygiene are critical to accomplishing SDGs, particularly SDG 3, Health and Wellbeing for All. To achieve this, greater attention to youth voices and perspectives, involving family and surrounding community, reliance on local cultural understandings of alcohol use and abuse, as well as health and wellness, is needed. Moreover, there is a need for an integrated multi-disciplinary approach to a practice involving social workers and other professionals such as psychiatrists, counsellors, and health workers to promote holistic responses. Given the consequences of harmful alcohol use on an individual’s worth and dignity and the more significant implications for family, community, and society, it is crucial to improve refugees’ access to substance use services. To comprehensively meet the needs of war-affected refugee youth, more substantial commitments are required for policymakers to work together with service providers because the knowledge, skills, and experiences of service providers are essential to effectively respond and address alcohol issues among refugee populations in precarious conditions. In the broader societal view, the socioeconomic and public health implications of harmful alcohol use among these war-affected refugee youth must be understood to inform relevant interventions at both individual and population levels, culturally and structurally competent policy solutions and appropriate livelihoods in resource constraint displacement environment.

## Data availability statement

The datasets presented in this article are not readily available because of ethical and confidentiality reasons. Requests to access the datasets should be directed to GM, godfrey.makoha@mail.mcgill.ca.

## Ethics statement

This study involved human participants. The McGill University Research Ethics Board 3 REB File#21-05-043 approved the study. The study was also conducted under the Office of the Prime Minister’s local legislation and institutional requirements. The studies involving humans were approved by the McGill University Research Ethics Board 3 REB File#: 21-05-043. The studies were conducted in accordance with the local legislation and institutional requirements. The participants provided their written informed consent to participate in this study.

## Author contributions

GM: project conceptualization, methodology, funding acquisition, data collection, analysis, writing, and editing. MD: paper co-conceptualization, writing, and editing. All authors contributed to the article and approved the submitted version.

## Footnotes

^1^The United Nations (UN) *Convention on the Rights of the Child* defines a child as “every human being below 18 years.” The UN defines youth as “those persons between the ages of 15 and 24 years.”
